# Establishing conditions for the generation and maintenance of estrogen receptor-positive organoid models of breast cancer

**DOI:** 10.1186/s13058-024-01798-6

**Published:** 2024-03-29

**Authors:** Michael U J Oliphant, Dipikaa Akshinthala, Senthil K. Muthuswamy

**Affiliations:** 1 Department of Cell Biology and Ludwig Cancer Center, Harvard Medical School, Boston, MA USA; 2 Laboratory of Cancer Biology and Genetics, Center For Cancer Research, National Cancer Institute, National Institute of Health, Bethesda, MD USA

## Abstract

Patient-derived organoid models of estrogen receptor-positive (ER+) breast cancer would provide a much-needed tool to understand drug resistance and disease progression better. However, the establishment and long-term maintenance of ER expression, function, and response in vitro remains a significant challenge. Here, we report the development of an ER+ breast tumor organoid medium (BTOM-ER) that conserves ER expression, estrogen responsiveness, and dependence, as well as sensitivity to endocrine therapy of ER+ patient-derived xenograft organoids (PDXO). Our findings demonstrate the utility of subtype-specific culture conditions that better mimic the characteristics of the breast epithelial biology and microenvironment, providing a powerful platform for investigating therapy response and disease progression of ER+ breast cancer.

**Supplementary Information:**

The online version contains supplementary material available at 10.1186/s13058-024-01798-6.

## Introduction

Breast cancer remains one of the most frequently diagnosed cancers and causes of cancer deaths among women worldwide [23]. Despite estrogen receptor-positive (ER+) breast cancer being the most prevalent subtype, the establishment of clinically relevant models remains a challenge. More recently, three-dimensional (3D) organoid models established from patient tumors bridge the gap between cell culture and patient-derived xenograft platforms [4, 9, 21]. Previous studies have shown that organoid media formulations are critical for the establishment, growth, and maintenance of patient-specific characteristics of ER+ breast cancer organoid models [9, 12, 21]. However, consistent establishment, decreased estrogen receptor (ER) expression, and loss of ER-dependent transcription after extended culturing continues to be a challenge for the generation and use of organoid models from ER+ breast tumors.

Here, we report the development of a simplified media considering breast tissue-relevant cytokines and growth factors to establish and expand ER+ organoids that retain ER expression over long-term culture. In addition, they retain responsiveness to estrogen and sensitivity to the anti-hormone therapeutic agent, fulvestrant, identifying a new approach for generating ER+ organoid models for breast cancer.

## Results

### Optimization of media conditions that support robust growth of ER+ PDX organoids

In designing the media, we excluded serum to prevent the proliferation of fibroblasts and stem cell factors to prevent reprogramming or the expansion of undifferentiated cells at the expense of differentiated epithelial cells. In particular, R-spondin-1 was not included due to its roles in promoting stem and basal epithelial cell differentiation and proliferation [14]. Noggin was not included for its role in promoting stemness by interfering with ER transcriptional responses [18]. Bovine pituitary extract (BPE) and B27 is used as a serum-free mitogenic supplement [10]  and to protect against oxidative stress, a common challenge that occurs in 3D cultures [15]. We included growth factors implicated in ER biology in developmental biology studies. For example, prolactin stimulates ER expression and works in concert with progesterone receptor to promote autocrine secretion-mediated mammary epithelial cell and breast cancer proliferation [6, 17]. Amphiregulin supports ER expression and estrogen signaling in the mammary gland [3]. IL6 expression is positively correlated with hormone receptor-positive tumors and has been shown to coordinate estrogen expression in vivo [5, 22, 24]. The above factors were added to DMEM/F12 media in conjunction with other growth factors frequently used to support the proliferation of mammary epithelial cells in culture and in vivo, including FGF2, FGF10, EGF, insulin, and hydrocortisone (See Additional file 1 for details of media recipe and preparation). Supplementation of estradiol to the media caused varying effects on cell growth; some organoid cultures showed no difference, whereas others showed a non-significant increase in growth (Additional file 1: Fig. S1). Furthermore, estrogen receptor expression and activity can be negatively regulated in both normal and cancer contexts during prolonged exposure to E2 in culture and in vivo ([1, 11]; hence no E2 supplementation was used for routine culture.

**Fig. 1:**
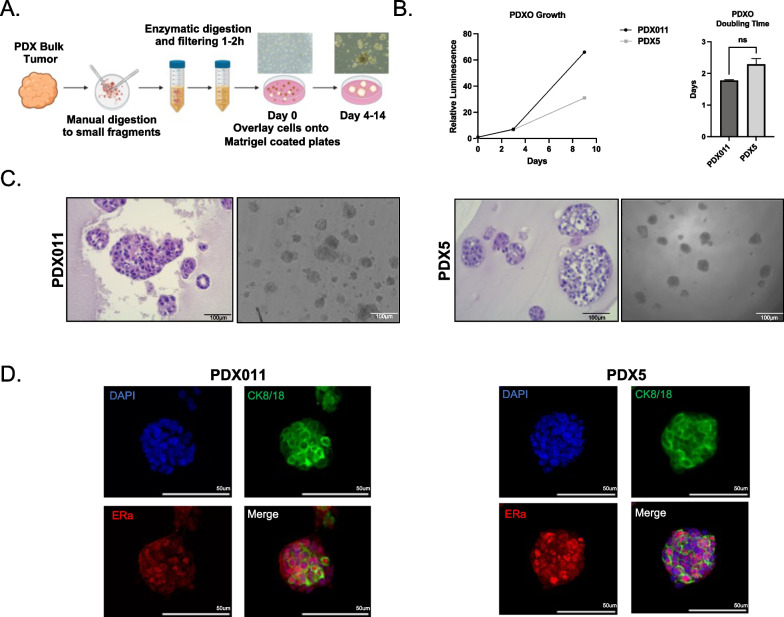
BTOM promotes robust growth and ER expression of ER+ PDXOs. **A** Schematic describing establishment of PDXOs. **B** A representative experiment of growth over time of PDX011 and PDX5 organoids, as measured by 3D Cell Titer Glo (Promega). Doubling time was calculated using GraphPad Prism Software. **C** H&E and brightfield images to show the morphology of established PDXOs. Scale bars: 100 μm. **D** Immunofluorescence of PDXOs displaying CK8/18 and ER-alpha expression. DAPI was used to stain nuclei

We used two independent ER+ patient-derived xenograft tumors [9] (Table 1) to find the optimal concentrations of the various growth factors to define a Breast Tumor Organoid Media for ER+ breast cancers (BTOM-ER) that support growth and expansion (Fig. 1a, Table 2). The PDXOs achieved robust growth with an average doubling time of 2.042 days (Fig. 1b) and remained stable in later passages. These data are consistent with the most commonly used ER+ breast cancer cell lines (T47D and MCF7), which have an average doubling time of 1–1.5 days [20, 25]. The phase and histomorphology of PDXOs were heterogeneous within cultures consisting of solid and hollow organoids with the formation of budding structures between 10 and 14 days post-plating (Fig. 1c). Both proliferation rates and phase morphological characteristics were relatively consistent over 15 passages, as determined by phase morphology and Incucyte live-cell imaging (data not shown). Importantly, immunofluorescence analysis for cytokeratin 8/18 and estrogen receptor demonstrated the presence of ER+ luminal tumor epithelia (Fig. 1d). Collectively, these data suggest that BTOM-ER allows for the establishment and maintenance of ER+ luminal epithelial organoid cultures that maintain proliferative capacity during long-term culture.

**Table 1:** PDXO Clinical information

PDX ID	ER	PR	HER2	ER mutation status	Additional genetic alterations	Morphology
PDX011	+	+	−	WT	N/A	Solid spheres, with branching for larger organoids
PDX5	+	+	+	L536P	RBM10 and RNF111 deletions	Solid spheres with small numbers of protruding cells

**Table 2:** Components of BTOM-ER growth media

	Supplements/growth factors	Vendor	Catalog #	Final concentration
*Reagent A*				
1	Bovine pituitary extract	Hammond cell tech	1078-NZ	0.8 ml for 100 ml
2	B27	Thermo	17504001	1.0 ml for 100 ml
3	Recombinant human FGF-basic (FGF2)	Peprotech	AF-100-18B	10 ng/ml
4	Recombinant human FGF10	Peprotech	100-26	10 ng/ml
5	Recombinant human EGF	Peprotech	AF-100-15	2.0 ng/ml
6	Recombinant human IL6	Peprotech	200-06	100 ng/ml
7	Recombinant human amphiregulin	Peprotech	100-55B	100 ng/ml
8	Recombinant human prolactin	Peprotech	100-07	10 ng/ml
9	Human insulin	Sigma	I2643-250MG	10 μg/ml
*Reagent B*				
	Hydrocortisone	Sigma	1316004-200MG	0.5 μg/ml

### PDXOs maintain estrogen receptor expression, are estrogen-responsive, and are sensitive to endocrine therapy

**Fig. 2:**
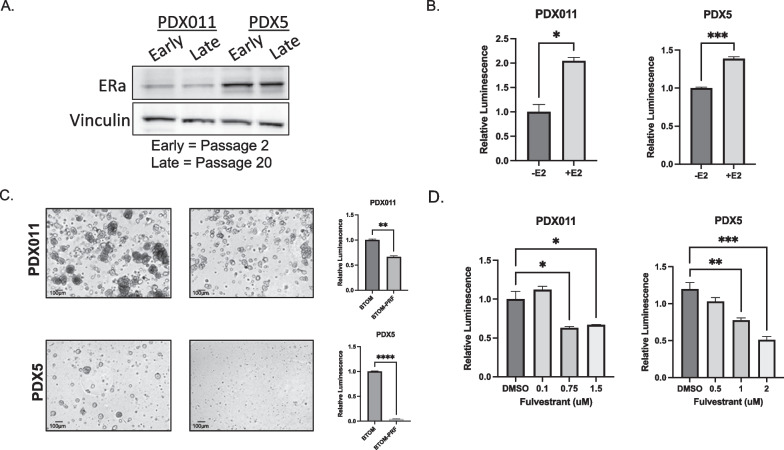
PDXOs grown in BTOM maintain estrogen-dependent phenotypes of ER+ breast cancer. **A** Western blot showing Estrogen Receptor alpha expression in PDX011 and PDX5 organoids. VINCULIN was used as a loading control. **B** Luminescence levels in PDX011 and PDX5 organoids after 10 days of treatment with b-estradiol or vehicle control (EtOH). N = 3. **C** Representative brightfield images of Day 10 PDX011 and PDX5 grown in either BTOM or phenol red-free BTOM (left). Luminescence values for PDX011 and PDX5 organoids 10 days after being grown in either BTOM or phenol red-free BTOM (N = 3). **D** Luminescence values for PDX011 and PDX5 after being treated for 5 days with increasing doses of fulvestrant or vehicle control (N = 3)

Consistent with ER expression detected in the PDX tumors in vivo, analysis of cell lysates from early (passage 2) and late passage (passage 20) organoid cultures expressed detectable ERalpha (Fig. 2a). Additionally, treatment of PDXOs with physiological levels of estrogen resulted in increased proliferation compared to vehicle control (Fig. 2b). In contrast, culturing the organoids in phenol red-free media to exclude the weak estrogenic activity of phenol red reduced cell proliferation in PDX5 tumor-derived cultures that are known to express mutant ER [9] (Fig. 2c). These results are consistent with previous studies that showed these PDXs are responsive to estrogen stimulation [8, 9].Organoid lines from PDX011 tumors expressing wild-type ER showed only a weaker but significant decrease in proliferation. Finally, we treated PDXOs with the selective estrogen receptor degrader (SERD) fulvestrant, which is a widely used treatment for ER+ breast cancer. As expected, fulvestrant treatment resulted in a significant decrease in proliferation compared to vehicle control, suggesting that PDXOs, like ER+ breast tumors, require ER function to proliferate (Fig. 2d). Thus, we demonstrate that ER+ PDXOs maintain ER expression, estrogen dependence, and responsiveness and retain endocrine sensitivity during long-term culture.

## Discussion

We report the development of an organoid culture media for the generation and maintenance of ER+ breast tumor organoids. We considered growth factors and cytokines with established roles in ER-mediated signaling and growth, as well as maintenance of ER expression, leading to the inclusion of components such as prolactin, amphiregulin, IL6, and bovine pituitary extract. We excluded regulators of stemness, including WNT, R-Spondin-1, and Noggin, to avoid the selection of less differentiated epithelial lineages at the expense of differentiated lineages. Given the consideration of growth factors that impact ER biology, it is likely BTOM-ER will be efficient in supporting the establishment and maintenance of ER+ luminal epithelial organoids from normal breast or mouse mammary glands; however, further studies will be required to investigate this possibility. We also recognize that the BTOM-ER we report here has multiple factors that could introduce confounding variables. However, we note that the factors used in the media were required to maintain ER expression and hence constitute an essential media for long-term expression of ER expression in primary breast tumor cells. It is possible that future efforts can identify strategies to simplify the media formulation. Nevertheless, we believe that, BTOM-ER represents a major advance for developing new clinically relevant models for ER+ breast cancer models that can be used for developing new therapies.

Similar to many other tumor organoid platforms, the organoid culture conditions outlined here do not contain fibroblasts or immune cells, which have been shown to play a significant role in ER-mediated transcription and endocrine therapy response [2, 19]. However, the culture conditions reported here are suitable for co-cultures, as we demonstrated recently for the culture of T cells with mouse or rat mammary tumor organoid models to investigate T-cell mediated killing of tumor epithelia [7, 16] to study the interaction between tumor-epithelial and stroma [12]. Thus, we believe that the culture conditions reported here can serve as a new platform for understanding ER biology in primary breast tumor-derived epithelia and for the development of ways to overcome drug resistance in ER+ breast cancer.

## Methods

### ER+ breast tumor organoid media (BTOM-ER)

For BTOM-ER growth media, 2.145 ml of Reagent A, 50 ml of Reagent B, and 1.0% penicillin–streptomycin were added to 100 ml of DMEM/F-12. Please see Table 1 for components of Reagent A and Reagent B and Additional file 1 for further details on components and media recipes.

### Establishment of PDX organoid cultures

PDX tumor tissue was placed in a 10 cm dish, and 1–5 ml of resuspension media (DMEM-F12, 1.0% Penicillin–streptomycin, and 1.0% BSA) was added to preserve the viability of the cells during processing. Tissue was then minced using sterile surgical scalpels into fragments ranging from 0.5 to 1.0 mm. Minced tissue was then transferred to a 15 ml conical tube and pelleted by centrifugation at 1500 rpm for 5 min at 4 degrees centigrade. After the supernatant was carefully removed, 2–10 ml of digestion media (DMEM-F12, 0.1 mg/ml Collagenase/Dispase, and 1.0% Penicillin–streptomycin) was added, and the tube was placed in a 37 degrees centigrade shaker for 30–90 min, with checking every 15 min to check progress and ensure proper digestion of the tissue. The tissue is then pelleted again and resuspended in Accutase for 15–30 min to digest tissue into organoid fragments further. The tissue is then put through a 250 mm strainer to remove debris or large tissue fragments while allowing organoids to pass through. Organoids are pelleted and gently resuspended in BTOM-ER containing 5% Matrigel and ROCK-inhibitor (10 mM Y267632, Tocris), and then plated on top of a solidified Matrigel-coated well. Media was changed every 3–4 days.

### Passaging of PDXOs

After the removal of BTOM-ER, digestion media is added to each well, and the plate is incubated at 37 degrees centigrade for 1.5 h. When the Matrigel has become a slurry and organoids are dispersed into small clumps or clusters, cold resuspension media is added at a 1:1 volume ratio to each well, and the suspension is transferred to a 15 ml conical tube. After centrifugation at 1500 rpm for 5 min, the supernatant is removed, and the pellet is resuspended in Accutase and incubated for 20–30 min at 37 degrees centigrade. After incubation, resuspension media is added to the suspension. After centrifugation and removal of the supernatant, the pellet is resuspended in BTOM-ER and plated as done during the establishment of cultures.

### Immunohistochemistry (IHC) and imaging of PDXOs

Immunohistochemistry was performed as previously described [13]. Briefly, organoid tissue sections were generated by plating 20,000 cells/well of an 8-chamber slide. After the formation of mature organoid structures (typically 7–10 days post-plating), organoids were fixed in 4% PFA for 2 h. After treatment with hematoxylin solution for 15 min, organoids were washed twice with water. The organoids were then scraped onto a solidified layer of histogel, with additional histogel added on top to create a sandwich within a cryomold. Once solidified, the histogel sandwich was transferred to a tissue cassette and fixed in 10% formalin for 16–24 h, followed by a brief wash in 70% EtOH. The cassettes were stored in 70% EtOH until submitted for sectioning. To obtain brightfield images of organoids in culture, cells were plated at a density of 25,000 cells/well, and images were taken at 20 ×, 10 ×, and 4 × magnification using Spot Imaging software.

### Proliferation and doubling time of PDXOs

Organoids were digested as above and treated with TrypLE instead of Accutase to generate single cells. Each assay well in a 96-well plate was pre-coated with 30 ml of Matrigel and was allowed to solidify in a 37 degrees centigrade incubator for at least 10 min before plating cell suspensions on top. Cells were resuspended in BTOM-ER at a density of 50,000 cells/ml, and 100 ml was added to each well in triplicate. On the indicated days, corresponding wells were assessed for viability using 3D Cell Titer Glo (Promega). Viability measurements were normalized to Day 0, and doubling time was calculated using GraphPad Prism software.

### Western blot analysis

Organoids were grown in six-well plates at a density of 250,000 cells/ml. Once organoids reached confluency, each well was washed with ice-cold PBS. Matrigel was broken up by pipetting, and the slurry was transferred into a 15 ml conical tube. An additional 1.0 ml of PBS was added to the well to collect any remaining organoids. Organoids were pelleted by centrifugation at 3000 rpm for 5 min, and the supernatant was removed. The pellet was resuspended with 1.0 ml of Cell Recovery Solution (Corning) and incubated for one hour on ice. The released organoids were pelleted by centrifugation, and the supernatant was removed. After washing the pellet once with ice-cold PBS, the cells were resuspended with RIPA buffer, and western blotting analysis was performed. Signals were detected using Amersham Imager System via chemiluminescence.

### Estrogen responsiveness and dependence of PDXOs

For both estrogen responsiveness and dependence, organoids were digested and plated as above 40–50,000 cells/ml, depending on the organoid line. Three days after plating, media was refreshed, and wells were treated with either 1.0 nM of b-estradiol or vehicle control (EtOH) using the Tecan D300e drug dispenser. For dependence, media was replaced with fresh BTOM-ER or phenol red-free BTOM-ER. Media was refreshed every 2–3 days. After 10 days, organoids were assessed for viability using 3D Cell Titer Glo (Promega). Viability measurements were analyzed using GraphPad Prism software.

### Endocrine therapy treatment of PDXOs

Organoids were prepared as above, and on day three, media was refreshed, and wells were treated with either vehicle control or indicated concentrations of fulvestrant (Selleckchem) using the Tecan D300e drug dispenser. Media was refreshed, and plates were retreated every 2–3 days. After 5 days of treatment, organoids were assessed for viability using 3D Cell Titer Glo (Promega).

### Statistics and reproducibility

Error bars were generated by Standard Error Mean (SEM) calculations. For experiments with two conditions, an unpaired one-tailed Student’s T-test was performed. For experiments with three or more conditions, one-way ANOVA followed by a Bonferroni comparison was used. Doubling time was calculated by applying a non-linear regression for exponential growth for each condition.

### Supplementary Information

**Additional file 1.** Supplementary material.

## Data Availability

Organoid lines generated will distributed freely upon publication of this manuscript.
