# Supplementary material for: Establishing conditions for the generation and maintenance of estrogen receptor-positive organoid models of breast cancer
Source: Breast Cancer Res. 2024 Mar 29;26:56. doi: 10.1186/s13058-024-01798-6 (PMC10979603; doi:10.1186/s13058-024-01798-6)
Supplement: Supplementary file 1 — Additional file 1. Supplementary material. [file 13058_2024_1798_MOESM1_ESM.docx]

**Supplementary Fig. 1**

**Suppl. Fig. 1: Effect of β-estradiol supplementation to growth media.**

Two organoid lines were grown in the presence of E2 (1.0 nM β-estradiol) or 1/10^th^ the concentration or no E2, and morphology was monitored by analysis of phase images over 12 days. A representative image from day 7 of the culture is shown.

**Supplemental Figure 2**

A


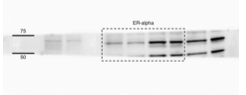


**B**

**
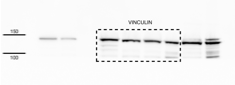
**

**Supplemental Figure 2:** Uncropped images of blots used in Figure 2A. A) uncropped image of ER alpha immunoblot. B) uncropped image of vinculin immunoblot.

Supplemental Material 1: Detailed methods for preparation of ER+ breast tumor organoid media

The initial organoid culture that develops after the plating of the processed tissue should be referred to as Passage 0 (P0). All subsequent passages should be serially numbered, and records maintained for each passage. We recommend freezing down early passage cultures before using them for experiments. We typically do not use cultures older than P20 for experiments for concerns related to culture-induced genetic drift.

- *We prepare master cocktails pre-mix and store them in small aliquots for extended periods of time (6 months).*
- *The master cocktail pre-mix is diluted in cell culture media with antibiotics and stored at 4 degrees for short-term use (~1 month).*
- *Just before every use, we prepare ‘growth media’ that contains 5% Matrigel.*

**Materials Needed**

| **Materials** | **Vendor** |  | **Catalog #** |
| --- | --- | --- | --- |
| Collagenase/Dispase(100  mg/ml) | Sigma |  | 11097113001 |
| Accutase | Sigma |  | A6964 |
| DMEM+F-12 | Thermo |  | 11330-032 |
| Rock Inhibitor (Y267632) | Tocris |  | 1254 |
| Matrigel (Growth factor | BD |  | 354230 |
| reduced) |  |  |  |
| (Concentration |  |  |  |
| ~8.5mg/ml) |  |  |  |
| Penicillin-Streptomycin  10,000 U/mL | Gibco |  | 15140-122 |
| Bovine Serum Albumin  Heat shock fraction | Sigma |  | A7906 |
| Growth Media | See recipe below |  |  |
| BTOM | See recipe below |  |  |
| Tissue strainer (250µm) | Thermo |  | 87791 |
| Chamber slides | BD or any vendor |  |  |
| Freezing media (Cryostar cs10) | Stem Cell Tech |  | 7930 |

**BTOM-ER Pre-mix cocktail recipe**

|  | **Supplements/Growth**  **factors** | **Vendor** | **Catalog #** | **Final**  **concentration** |
| --- | --- | --- | --- | --- |
| **Reagent A – (**See note below) | | | | |
| 1 | BPE | Hammond cell  tech | 1078-NZ | 0.8 ml for  100ml |
| 2 | B27 | Thermo | 17504001 | 1.0 ml for  100ml |
| 3 | Recombinant Human FGF- Basic (FGF2) | Peprotech | AF-100- 18B | 10ng/ml |
| 4 | Recombinant Human  FGF10 | Peprotech | 100-26 | 10ng/ml |
| 5 | Recombinant Human EGF | Peprotech | AF-100-15 | 2ng/ml |
| 6 | Recombinant Human IL6 | Peprotech | 200-06 | 100ng/ml |
| 7 | Recombinant Human  Amphiregulin | Peprotech | 100-55B | 100ng/ml |
| 8 | Recombinant Human  Prolactin | Peprotech | 100-07 | 10ng/ml |
| 9 | Human Insulin | Sigma | I2643- 250MG | 10ug/ml |
| **Reagent B – (**See note below) | | | | |
|  | Hydrocortisone | Sigma | 1316004-  200Mg | 0.5ug/ml |

### **Notes:**

Aliquoting B27.

1. Place the frozen B27 stock in a 4-degree fridge overnight.
2. On the second day, gently invert the fully thawed B27 a few times and aliquot into 2.0 ml aliquots.
3. Store the tubes in a -80 degree freezer.

Aliquoting BPE

1. Place the frozen BPE in a 4-degree fridge overnight to thaw.
2. Incubate the fully thawed BPE in a 37-degree water bath for one hour. This helps lipids in BPE to dissolve.
3. Transfer the 100 mL BPE into two 50 mL conical tubes and centrifuge at 2500 rpm for 5 minutes.
4. Use a 10 mL pipette to transfer the supernatant into two new 50 mL conical tubes. Discard the precipitates.
5. Aliquot BPE into 2.0 ml microcentrifuge tubes and store in a -80 freezer.

### Growth Factors:

### Prepare growth factor stock solutions as 1000x stocks following per manufacturer’s recommendation and store them in 100 ml aliquots.

### Hydrocortisone:

### Prepare a 1000x stock and store it in 100 μl aliquots.

# Media Recipes:

**Digestion Media:**

DMEM/F12

1:100 dilution of 100mg/ml stock Collagenase/ Dispase

1.0 % Penicillin-streptomycin

Preparation Tip: Prepare only the needed amount, fresh just before use, and discard the unused portion of the media.

**Resuspension Media:**

DMEM/F12

1.0 % BSA

1.0 % Penicillin-Streptomycin

Preparation Tip: Add 1% BSA by weight to DMEM+1.0% Penicillin- streptomycin-containing media and stir to dissolve the BSA. Once dissolved, filter sterilize using a 0.2µ filter and store at 4^o^C.

**BTOM-ER Growth Media:**

DMEM/F-12: 100 ml

2.145 ml of Reagent A

50 ml of Reagent B

1.0% Penicillin-Streptomycin

Preparation Tip:

*Do NOT mix Reagent A and Reagents B by themselves as the alcohol in Reagent B will denature the growth factors*

After adding all ingredients, filter the media through a 0.2µ filter

Store prepared media at 4^0^C for NO more than one month.

**Culture Media (make fresh for immediate use):**

BTOM-ER growth media.

5.0 % GFR-Matrigel

10 µM Y267632, Rock inhibitor (10mM, a 1000x stock). Rock inhibitor is prepared in sterile Phosphate Buffered Saline.

Preparation Tip: Prepare fresh. You may premix BTOM-ER growth media and ROCK inhibitor and keep them on ice. Add Matrigel just before use. Discard unused portions of the media.

**Freezing Media:**

Cryostar freezing media

10 µM Y267632, Rock inhibitor

**Supplementary Materials 2: Detailed Methods**

**Establishment of PDX organoid cultures**

PDX tumor tissue was placed in a 10 cm dish, and 1-5 ml of resuspension media (DMEM-F12, 1.0 % Penicillin-streptomycin, and 1.0 % BSA) was added to preserve the viability of the cells during processing. Tissue was then minced using sterile surgical scalpels into fragments ranging from 0.5-1.0 mm. Minced tissue was then transferred to a 15ml conical tube and pelleted by centrifugation at 1500 rpm for 5min at 4 degrees centigrade. After the supernatant was carefully removed, 2-10 ml of digestion media (DMEM-F12, 0.1mg/ml Collagenase/Dispase, and 1.0% Penicillin-streptomycin) was added, and the tube was placed in a 37 degrees centigrade shaker for 30-90 min, with checking every 15 min to check progress and ensure proper digestion of the tissue. The tissue is then pelleted again and resuspended in Accutase for 15-30 min to digest tissue into organoid fragments further. The tissue is then put through a 250 mm strainer to remove debris or large tissue fragments while allowing organoids to pass through. Organoids are pelleted and gently resuspended in BTOM-ER containing 5% Matrigel and ROCK-inhibitor (10 μM Y267632, Tocris), and then plated on top of a solidified Matrigel-coated well. Media was changed every 3-4 days.

**Passaging of PDXOs**

After the removal of BTOM-ER, digestion media is added to each well, and the plate is incubated at 37 degrees centigrade for 1.5 hours. When the Matrigel has become a slurry and organoids are dispersed into small clumps or clusters, cold resuspension media is added at a 1:1 volume ratio to each well, and the suspension is transferred to a 15ml conical tube. After centrifugation at 1500 rpm for 5 min, the supernatant is removed, and the pellet is resuspended in Accutase and incubated for 20-30 min at 37 degrees centigrade. After incubation, resuspension media is added to the suspension. After centrifugation and removal of the supernatant, the pellet is resuspended in BTOM-ER and plated as done during the establishment of cultures.

**Immunohistochemistry (IHC) and Imaging of PDXOs**

Immunohistochemistry was performed as previously described (Huang et al., 2020). Briefly, organoid tissue sections were generated by plating 20,000 cells/well of an 8-chamber slide. After the formation of mature organoid structures (typically 7-10 days post-plating), organoids were fixed in 4% PFA for 2 hours. After treatment with hematoxylin solution for 15 minutes, organoids were washed twice with water. The organoids were then scraped onto a solidified layer of histogel, with additional histogel added on top to create a sandwich within a cryomold. Once solidified, the histogel sandwich was transferred to a tissue cassette and fixed in 10% formalin for 16-24 h, followed by a brief wash in 70% EtOH. The cassettes were stored in 70% EtOH until submitted for sectioning. To obtain brightfield images of organoids in culture, cells were plated at a density of 25,000 cells/well, and images were taken at 20x, 10x, and 4x magnification using Spot Imaging software.

**Proliferation and Doubling Time of PDXOs**

Organoids were digested as above and treated with TrypLE instead of Accutase to generate single cells. Each assay well in a 96-well plate was pre-coated with 30 ml of Matrigel and was allowed to solidify in a 37 degrees centigrade incubator for at least 10 min before plating cell suspensions on top. Cells were resuspended in BTOM-ER at a density of 50,000 cells/ml, and 100 ml was added to each well in triplicate. On the indicated days, corresponding wells were assessed for viability using 3D Cell Titer Glo (Promega). Viability measurements were normalized to Day 0, and doubling time was calculated using GraphPad Prism software.

**Western Blot Analysis**

Organoids were grown in six-well plates at a density of 250,000 cells/ml. Once organoids reached confluency, each well was washed with ice-cold PBS. Matrigel was broken up by pipetting, and the slurry was transferred into a 15 ml conical tube. An additional 1.0 ml of PBS was added to the well to collect any remaining organoids. Organoids were pelleted by centrifugation at 3000 rpm for 5 min, and the supernatant was removed. The pellet was resuspended with 1.0 ml of Cell Recovery Solution (Corning) and incubated for one hour on ice. The released organoids were pelleted by centrifugation, and the supernatant was removed. After washing the pellet once with ice-cold PBS, the cells were resuspended with RIPA buffer, and western blotting analysis was performed. Signals were detected using Amersham Imager System via chemiluminescence.
